# Positive Psychology in Therapeutic Songwriting for People Living with Late-Life Depression—An Intervention Protocol

**DOI:** 10.3390/brainsci12050626

**Published:** 2022-05-10

**Authors:** Jasmin Eickholt, Felicity A. Baker, Imogen N. Clark

**Affiliations:** 1 Creative Arts & Music Therapy Research Unit, Faculty of Fine Arts and Music, University of Melbourne, Melbourne, VIC 3006, Australia; felicity.baker@unimelb.edu.au (F.A.B.); imogen.clark@unimelb.edu.au (I.N.C.); 2 Faculty of Applied Social Sciences, University of Applied Sciences Würzburg-Schweinfurt, 97070 Würzburg, Germany; 3 Centre for Research in Music and Health, Norwegian Academy of Music, 0010 Oslo, Norway

**Keywords:** therapeutic songwriting, music therapy, positive psychology, depression, wellbeing, older people, late life

## Abstract

(1) Background: An increasing number of people are living with late-life depression, yet non-pharmacological treatments to help manage symptoms are limited. Two interventions, positive psychology and music therapeutic songwriting, have independently led to decreased depressive symptoms and an improved wellbeing in older people over 65 years old. (2) Methods: This article describes the development of a therapeutic songwriting program for people living with late-life depression. Knowledge from positive psychology and therapeutic songwriting was combined to maximize the potential benefits. (3) Results: The intervention program has ten weekly 45 min sessions that incorporate elements from positive psychology into therapeutic songwriting. Using a three-song approach encompassing ongoing musical practices, different positive psychology interventions were incorporated to support the experiences associated with a flourishing life. The intervention protocol for older people presented here is distinct from previous deficit-orientated approaches in that it shifts the focus to positive experiences, resources, and the individual’s ability to decrease their own depressive symptoms and improve their wellbeing. (4) Discussion: This protocol presenting a therapeutic songwriting program meets the need to develop new non-pharmacological treatment options. However, further studies are needed to examine the feasibility and impact of the intervention program on late-life depression and wellbeing in older people.

## 1. Introduction

The number of people living with late-life depression continues to increase. It is estimated that by 2050 there will be almost 1.9 billion individuals over 60 years old living with late-life depression [1]. In 2013, the National Centre for Health Statistics (NHANES) reported that 61.7% of people over 65 years old experience at least one depressive symptom [2]. At the same time, the effects of traditional therapies are proving unsuccessful for an increasing number of older people. Hence, there is a need to find successful evidence-based treatments for people living with late-life depression [1].

Positive psychology is one treatment option. It presents a psychological orientation that turns the focus from deficits related to diseases to strengths, virtues, and the building of positive qualities. Positive psychology acknowledges and values subjective experiences and recognizes happiness, wellbeing, and satisfaction, as well as hope and optimism [3]. Wellbeing is associated with two aspects—hedonic and eudaimonic wellbeing. Hedonic wellbeing includes happiness with an optimal balance between positive and negative experiences and perceived life satisfaction [4]. The main goal of hedonic wellbeing is to experience pleasure [5]. Conversely, eudaimonic wellbeing is about living in accordance to one’s own values, self-realization, and meaning [5], emphasizing long-term wellbeing [6]. Eudaimonic and hedonic wellbeing are presented in Seligman’s [7] ‘Well-Being Theory’, seeking to support the experiences associated with a flourishing life [4,6]. His theory emphasized five measurable elements (acronym PERMA) of positive psychology that can lead to wellbeing and flourishing. These are: experiencing positive emotion (P), more engagement (E), good relationships (R), accomplishment (A), and meaning (M). Meaning is understood as the “belonging to and serving something that you believe is bigger than the self” [7] (p. 45), and is independent of positive emotion, engagement, relationships, and accomplishment, which are mainly experienced within relationships. Character strengths are pathways towards each of the PERMA elements, and ultimately towards an improved wellbeing [7].

The effects of positive psychology interventions on subjective experiences, psychological wellbeing, and depression were investigated in a meta-analysis by Bolier, Haverman [8]. In 40 included randomized studies (6319 adults), small-to-medium effect sizes (Cohen’s *d*) for positive psychology interventions were observed for depression (*d* = 0.7), subjective wellbeing (*d* = 0.43), and psychological wellbeing (*d* = 0.41) [8]. Two of these studies examined different positive psychology interventions that included samples with some participants over 65 years old. In these studies, the ages ranged from 22–77 years old (*n* = 65 [9]) to 18–79 years old (*n* = 165 [10]). A further two studies were conducted with a population of older adults with a mean age of 72.9 (*n* = 193 [11]) and 64.3 years old (*n* = 142 [12]). In other studies, significant improvements in depressive symptoms, psychological outcomes (such as life satisfaction and mood), and social wellbeing were observed following positive psychology interventions for older people [13,14,15,16,17,18,19]. Unfortunately, data from some of these studies were missing, making it impossible to calculate the effect sizes. The reported effect sizes ranged from small to large [13,14,15,16,17,18,19]. Music therapy can provide a highly promising non-pharmacological intervention for late-life depression. Therapeutic songwriting supports the songwriter (in this article, the term songwriter is used for the patient/client) to express themselves, release stress, and cope with the experiences they encounter in their everyday lives [20,21]. The songwriting helps them to develop a sense of self and to facilitate clarifying and externalizing their thoughts, fantasies, and emotions. A written song can thereby become a powerful reminder of their inner sources and strengths as well as their accomplishments [20].

Different authors have suggested the effectiveness of therapeutic songwriting in adults living with depression. However, based on the review findings to date, just nine studies have investigated therapeutic songwriting in depression ([22,23,24,25,26,27,28,29,30]). Three investigated therapeutic songwriting for older people, with observed medium- (Cohen’s *d*; *d* = 0.64; *d* = 0.57 [23,26]) to-large effect sizes (*d* = 0.83 [24]), suggesting a reduction in depressive symptoms. However, there is a lack of large randomized controlled studies investigating the effects of therapeutic songwriting.

The aim of this study is to develop a therapeutic songwriting program that addresses the need to develop new successful treatments. In combining knowledge from positive psychology and therapeutic songwriting, the developed intervention program aims to support the management of depressive symptoms and to improve wellbeing in older people living with late-life depression.

## 2. Materials and Methods

The development of the intervention program was informed by the new Medical Research Council guidance [31]. The guideline describes four key elements that are important for developing and evaluating complex interventions. The development, as first described as a key element, concerns the identification of the existing evidence, the development of a theory that informs the intervention, and the determination of the intervention design. Other key elements are feasibility and pilot studies, and the evaluation and the implementation of the intervention [31]. The following paper presents the results of the ‘development’ phase.

Elements of positive psychology with evidence demonstrating decreased depressive symptoms and increased wellbeing were incorporated into a therapeutic songwriting program. We sought to integrate hedonic and eudaimonic happiness into the intervention program presented here by using all the elements of the PERMA model.

In combining therapeutic songwriting and positive psychology, the songwriter should receive the benefits of both disciplines, accelerate and intensify the reduction in depressive symptoms, and enhance their wellbeing. The program was further influenced by knowledge from music therapy and different psychological orientations such as music psychology and behavioral therapy. The presented case studies in this article were conducted in Germany with participants from the Würzburg region.

The program is divided into three parts, each with a different therapeutic focus and each including both positive psychology and therapeutic songwriting components. During the final session (session 10), the songwriter is asked to reflect on what was learned during the therapeutic process.

## 3. Results

### 3.1. Overview

**Figure 1:**
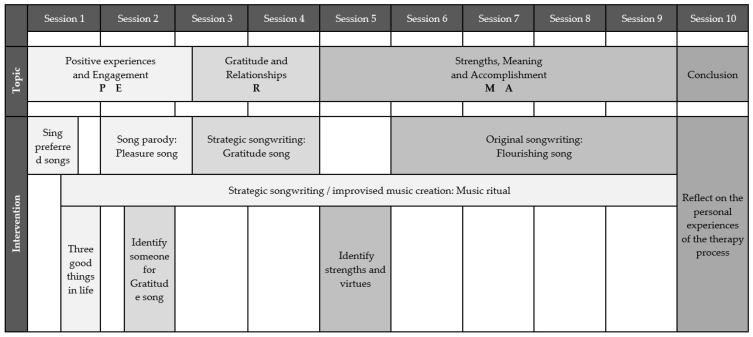
Overview focus and applied intervention of each session.

The overall aim of the intervention program is to decrease depressive symptoms and improve wellbeing by incorporating all facets of the PERMA model: positive emotion, engagement, relationships, meaning, and accomplishment. The ten-session individual therapy intervention is offered once a week for 45 min (a more detailed explanation of the program, including more songwriting techniques and background information will be included in a dissertation of the first author, which will be available through the University of Melbourne or by contacting the first author). If a session cannot take place at the scheduled time, the music therapist and songwriter can reschedule to the next available time, and at the very latest, to the regular scheduled time the following week. When this occurs, the duration of the entire program is extended until all ten sessions have been implemented. The songwriter is asked to complete three homework activities that take up to five minutes per day. If these tasks are not completed by the songwriter, this is discussed and completed with the music therapist at the beginning of the following session. Figure 1 details the focus and intervention content of each session.

The first part of the program asks songwriters to focus on positive experiences and engagement. The ‘Pleasure song’ is written during sessions two and three with an extension to a music ritual that frames positive experiences into short musical phrases in each session. Starting with a song parody (the ‘Pleasure song’), the songwriter offers new lyrics to a known precomposed song [20]. The second part (sessions 4 to 5) focuses on gratitude and relationships. Here, a precomposed strategic songwriting approach uses an unfamiliar melody [20] to write lyrics for a ‘Gratitude song’. The third and final part of the program (sessions 6 to 9) focuses on strengths, meaning, and accomplishment. A ‘Flourishing song’ is written with an original songwriting approach that involves the creation of lyrics and music by the songwriter, with support from the music therapist [20].

**Figure 2:**
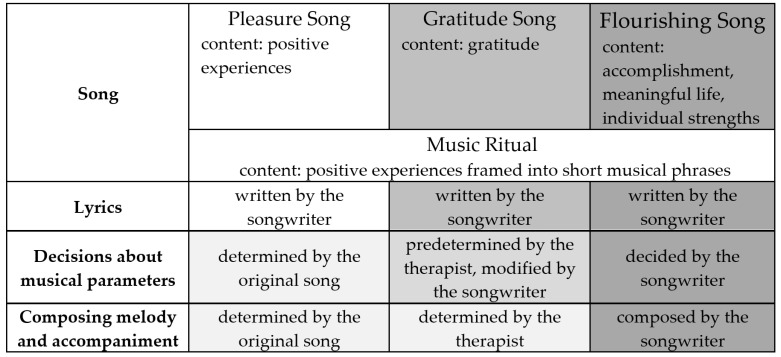
Elements of songwriting procedure.

The level of difficulty of the applied songwriting techniques increases as the intervention process progresses. The songwriter is first introduced to the concept of song creation and, as the sessions progress, more and more songwriting elements are incorporated with the support of the music therapist. Figure 2 shows the songwriting elements that are prepared by the music therapist or determined by the songwriter, depending on the status of the intervention progress. The ‘Pleasure song’ is based on an existing song selected by the songwriter and there are less opportunities to change the musical parameters (such as the rhythm or melody). The musical parameters of the ‘Gratitude song’ are predetermined by the music therapist by providing various precomposed songs to choose from. These songs are informed by the songwriter’s background and interests following an assessment in session one and ongoing throughout the intervention process. The music therapist demonstrates different options for changing the musical parameters. The songwriter chooses the parameters that they prefer and identify with the most. Finally, the original songwriting technique of the ‘Flourishing song’ enables the songwriter to choose the musical parameters from the beginning. For this ‘Flourishing song’, the music therapist does not provide any predetermined musical parameters but provides the songwriter with as many or as few musical suggestions needed to support their creation of an original song.

The songwriting process can be adjusted to the needs of the songwriter, such as to their interests, cognitive and musical/writing abilities, or context (for example, where time is limited). To promote a sense of accomplishment within each session and in the overall process, the music therapist supports the songwriter to ensure that the individual components of and then the final versions of the songs are completed within each of the ten sessions.

### 3.2. Part 1—Session 1–3 ‘Pleasure Song’

The first part introduces the songwriter to the intervention program and represents the positive emotion (P) and engagement (E) components of the PERMA model. The aim of the first and second part is to:

- Broaden perception (such as mental flexibility and creativity);
- Build resilience and social, intellectual, and emotional resources;
- Improve social engagement, relationships, interaction skills, and sense of belonging;
- Promote brain activation and autobiographic memories;
- Promote an awareness of self and one’s musical identity;
- Link music with positive emotions.

Before the first song is written, the songwriter and music therapist sing or listen to songs that evoke positive emotion to build rapport. Songs can be a good starting point for musical interaction and songwriting. The music that evokes positive memories of the past and motivate the songwriter to share their own stories and to stimulate autobiographical recall [20,32] is selected by the songwriter. Providing the songwriter with songbooks that list different topics facilitates finding songs that are already linked to positive experiences.

The songwriter is further informed about the impact of positive emotions and engagement. Positive emotion and the regulation of negative emotion enhances positivity and optimism and has been found to reduce anxiety, depressive symptoms, frailty, and mortality rates in old age [13,33]. Being engaged with a task can lead to ‘flow-experiences’. Flow experiences are described as the feeling of being completely absorbed in a task that is challenging but achievable. They lead to personal growth and the gaining of higher capacities [34].

#### 3.2.1. Music Ritual

For the music ritual and the first song, the songwriter, guided by the music therapist, brainstorms positive experiences that happened to them in recent days. To facilitate deeper insight, these positive experiences are then shaped into a short musical phrase that becomes a music ritual for each subsequent session. To enhance the musical connection, the music therapist offers a short and easily memorized melody and accompaniment that incorporates the songwriter’s preferred music style. During the program, the music therapist continually tries to motivate the songwriter to improvise and/or create new melodies on their own. In improvised music creation, the lyrics and melody of the songs are created ‘in the moment’ rather than over time (as occurs with brainstorming) [20] (p. 132). This music ritual takes place at the beginning of each subsequent session to train the awareness of positive experiences.

Case study example: In the first session, a 71-year-old woman described her experiences over recent days. It was difficult for her to identify good things. However, together with the music therapist, she was able to describe events that were positive. She had enjoyed going to the city to buy an air freshener. She had been doing this regularly. The first musical phrase was “On this day, I was in the city. Smell of lavender, smell of vanilla, I like that so much” (translated by the author). In the following sessions, it was still difficult for her, but she needed less time to identify good things. Her time with other people was often reported and recognized as being very important for her. At the end of the intervention, her description of good things gained depth and became more abstract. Her last musical phrase was: “I get support when it gets difficult. Then I feel relieved and can finally look forward” (translated by the author).

#### 3.2.2. ’Pleasure Song’

**Figure A1:**
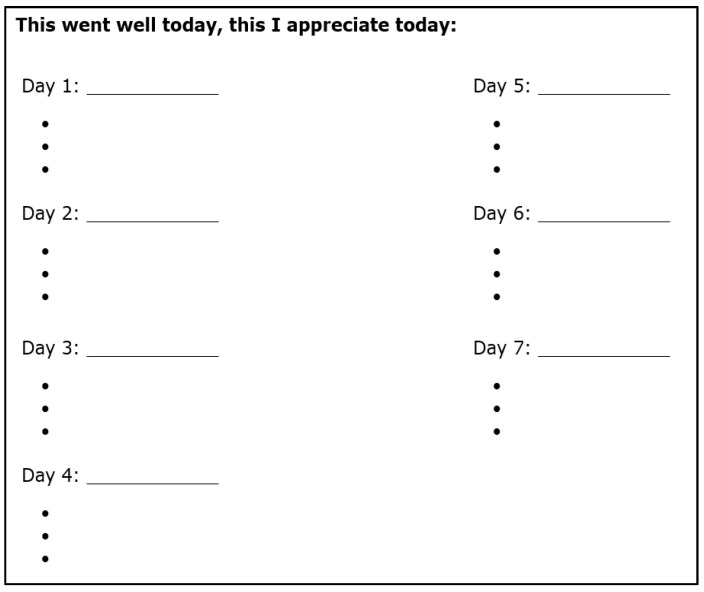
Worksheet for the ‘Pleasure song’.

The first song is the ‘Pleasure song’. The songwriter is introduced to the positive psychology intervention ‘three good things in life’, where they write down three good and successful things per day for one week. This positive psychology intervention has already demonstrated positive effects on depression and happiness among adults [35]. A worksheet can be used to write these positive experiences (see Figure A1 in Appendix A). The worksheet also serves as a reminder for the task. In session two, the songwriter’s collected positive experiences are categorized into areas that evoke pleasure.

The steps of the therapeutic songwriting approach include the verbal introduction of the songwriting process, with a short musical example of the rewritten lyrics of a song/song part by the music therapist. Then, the songwriter can choose one of the songs that are already linked to positive emotions for the first song. As opposed to the “Song parody” therapeutic songwriting technique where the songwriter rewrites parts of or the whole lyrics of the existing song and the music therapist can offer the ‘song transformation’ method, here, the songwriter may also vary the melody and accompaniment if they wish [36]. This songwriting approach allows the songwriter to just focus on the lyrics without creating the music and allows for a gradual introduction to writing songs.

The ‘experiences that lead to pleasure’ topic is explored by the results of the ‘three good things in life’ positive psychology intervention. The collected experiences are allocated to areas or a conclusion, as appropriate. This information is then allocated to the given song structure and the songwriter starts to shape new lyrics on the melody of the pre-existing song. The finished song can be sung and refined, if required. A record of the song acts as a reminder of the exploration of the topic. At least one written song (and/or song part) is repeated at the beginning of each session.

Case study example: An 87-year-old woman collected positive experiences that were related to different activities provided by a residential care facility and in nature. Every morning, she sat in front of a window to admire how nature changed with the seasons. Mindfulness and gratefulness were very important for her. She chose a strophic song and allocated the two categories to two verses. The lyrics of the one verse were “I always keep my eyes open, for the beauty, the things of nature. I see the colors of the leaves, and they soar through the air” (translated by the author). She expressed feeling proud about the song and enjoyed showing the song to other residents in the therapeutic singing group.

### 3.3. Part 2—Session 3–4 ‘Gratitude Song’

The second part focusses on the relationships (R) concept of the PERMA model. At the end of session three, a person or spiritual/symbolic figure is determined for the second song. The basis of the song is the positive psychology intervention ‘gratitude visit’, which has demonstrated positive effects on happiness and depression among adults [35]. The ‘Gratitude song’ is written to address someone specific from the songwriter’s past and/or present life. This person is someone who has impacted the songwriter in a positive way and who they never properly thanked. In comparison to the original songwriting, no letter must be delivered to the person. Instead, the song might be written for a living or deceased person, a group of people (such as their family), or for spiritual/symbolic figures. In the strategic songwriting approach, precomposed music is provided by the music therapist and meets the musical interests and needs of the songwriter. This songwriting technique allows the songwriter to keep the focus on writing lyrics but for an unknown song. In addition, they still have the opportunity to change some musical parameters, such as the melody, accompaniment, and harmonic progressions. A song that meets the preferred style of the songwriter gives more meaning to the song [20].

**Figure A2:**
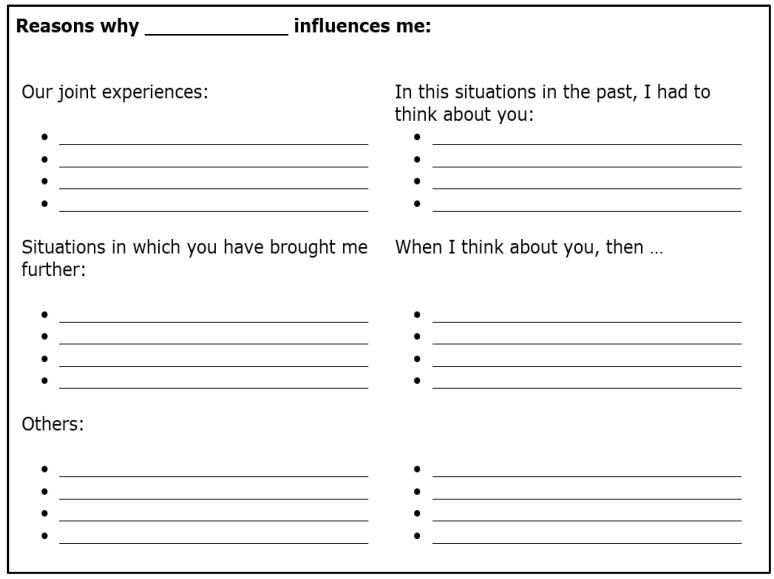
Worksheet for the ‘Gratitude song’.

For the song, the songwriter collects information about the person or spiritual/symbolic figure. This might be special situations or general traits the songwriter is grateful for. The collection takes place within and after session three. A worksheet can support the collection by asking, for example, for joint experiences (see Figure A2 in Appendix A). In session four, the music therapist presents possible precomposed songs. The songwriter chooses the song that best matches their interests, needs, and the statement of the collected information. The song can thereby have a traditional song structure with verses and a chorus, or it can be a strophic song without a chorus. In a strophic song, the lyrics continue, and one line can contain a main statement that is repeated in every verse to intensify the statement if desired. Strophic songs are appropriate where a story is told and a chorus would interrupt the flow of the song [37]. The 12-bar blues is one form of a strophic song that can be effective for writing songs with people with depression [29,30]. In the next step, the songwriter writes the lyrics based on the collected information, sings the song, and refines the lyrics and musical components, if required. The finished song is recorded with the songwriter. This can take place at the beginning of session five.

Case study example: For an 83-year-old man, it was important to show gratitude to his family but to also recognize God’s goodness. In the chorus, he wrote: “I have been lucky in my life, with family and God’s blessing […] I don’t want to give up the good times” (translated by the author). Then he wrote one verse about the kindness and support of his deceased wife, further acknowledging her as a good mother and grandmother. A second verse was about his children and grandchildren, acknowledging their good relationship. He enjoyed showing the song to them.

### 3.4. Part 3—Session 5–9 ‘Flourishing Song’

The third part focusses on the meaning (M) and accomplishment (A) concepts of the PERMA model. The aim of this part is to recognize the songwriter’s virtues and strengths, and how these traits were used to achieve their accomplishments and to achieve a meaningful life. Recognizing one’s strengths can improve wellbeing and decrease depression. It is connected with life satisfaction and supports the songwriter to cope with stress and trauma. Emphasizing the songwriter’s strengths, potentials, and resources can increase their confidence, competence, self-esteem, and resilience. Further, the focus on their strengths supports the building of effective coping strategies, a sense of empowerment, and a more integrated self-concept [20,38].

#### 3.4.1. Identifying Strengths

Together with the music therapist, individual strengths and situations where the songwriter’s strengths were used in the past are explored. The strengths could be used for their own achievements but also to improve someone else’s life. In addition, previously reported experiences can be reflected in this discussion. After the initial brainstorming, an overview of their virtues and strengths (see Table 1) is offered, based on the Values in Action Inventory of Strengths [39], to further support the identification of the songwriter’s strengths. The identified strengths can be written down on post-it papers and collected on a blank sheet to symbolize the abundance of strengths of the songwriter. After identifying the individual strengths, signature strengths are determined by asking for strengths that stand out in the songwriter’s personality. Identifying and using signature strengths in a new way can lead to increased happiness and decreased depression [14,35].

**Figure A3:**
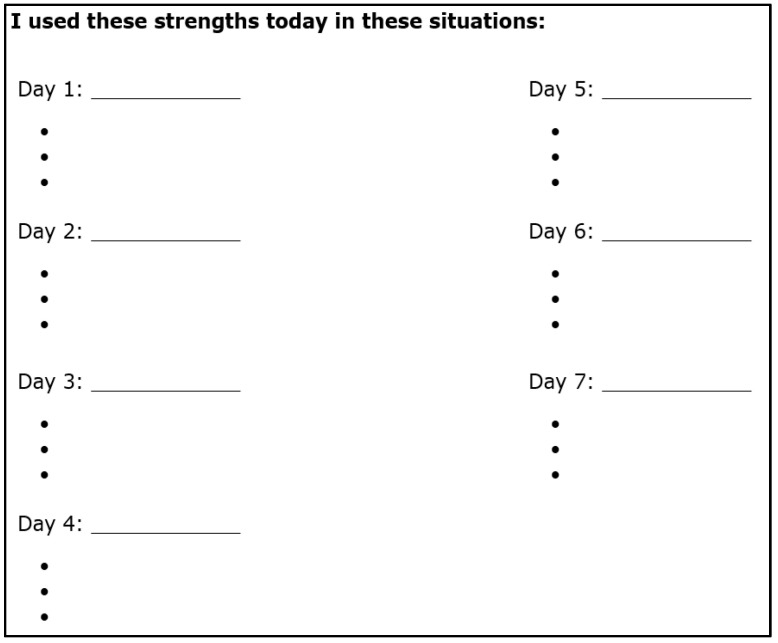
Worksheet for identifying strengths.

Together with the music therapist, the songwriter finds examples where their own signature strengths are already used in their everyday life, and they start to understand how their signature strengths could be used in a new way. Using their signature strengths in a new way is a positive psychology intervention that has demonstrated positive effects on happiness and wellbeing among adults [35]. Between the sessions, the songwriter is asked to write down which strengths were used and in which situations and day of one week. For this purpose, an overview of the songwriter’s strengths and a worksheet for writing down the strengths and situations are provided (see Figure A3 in Appendix A). Asking for different situations where the songwriter experienced their identified strengths can intensify the experience of their strengths and further support the collection of potential information for the lyrics of the ‘Flourishing song’.

Recognizing one’s strengths can be difficult for older people living with depression. An 83-year-old man reported that he thought he had no strengths. Through the intervention, however, he could recognize them. An 83-year-old woman reported that she did not recognize her own strengths in her daily life. She explained that they were more likely to be perceived in her subconscious.

#### 3.4.2. ’Flourishing Song’

The ‘Flourishing song’ is about what a meaningful and accomplished life would look like. A strong sense of meaning correlates with wellbeing, quality of life, and life satisfaction, whereas a lack of life satisfaction can lead to depression and social withdrawal. The song can include what the songwriter is proud of, what is important for them, or what they have already done to achieve meaning and accomplishment, using their own strengths. In addition, wishes and upcoming plans can be integrated.

For the brainstorming, a guiding question might be: ‘What is important in your life that gives you a sense of a meaningful life? Is there something you want to leave to the persons around you, for example virtues, or immaterial and material possessions?’ Then, individual ways of experiencing a meaningful life using signature strengths are discussed. For the song, all previously reported experiences that guided the songwriter to experience a flourishing life can be shaped into the lyrics (for example, experiences reported during the music ritual; see part 1). They might, for example, be experiences that influence the songwriter’s life in the present and for which the songwriter is grateful for (see part 2), or simply experiences that relate to the songwriter’s individual traits (see part 3).

After brainstorming about strengths, achievements, and what a meaningful life looks like, the song is written using an original songwriting approach. All information can be allocated to a song structure. A main statement, conclusion, or appeal can be stated in the chorus. The chorus can be used as a starting point for writing lyrics and music. A song structure might be as follows:

- Verse 1: what were the achievements in the songwriter’s past?
- Verse 2: what is meaningful for the songwriter in the past and present?
- Verse 3: what does the songwriter want to achieve in the future?
- Chorus (examples):
  ○ Who is the songwriter and what are their signature strengths, traits, and attitudes?
  ○ What does the songwriter want to leave to the persons around?
  ○ What is a guiding slogan for the songwriter?
  ○ What does the songwriter want to tell the people around them (e.g., memories or appeal)?

In the next step, the lyrics and music for verses and other elements are created. After the refinement of the lyrics and music, the song is recorded for the songwriter.

Case study example: For the ‘Flourishing song’, an 82-year-old man explored his life achievements with the music therapist, including his strengths and what he believed was important to him in life. In the past he was a very successful winery owner. He had always worked hard. This was used for the first verse. It was also important to him that he could share his knowledge and success with his son. He was very proud of his son’s successes. This was presented in the second verse. Work and success were very important for the man. However, he summarized that satisfaction is most important in life and that he is grateful for every new day. This summary was presented in the chorus: “Thank you for this life, for every new day […] Satisfaction is the greatest thing I have” (translated by the author). During the therapy, he revealed how challenging it was to deal with the lack of contact with his son. In writing the song, he was motivated to get in contact with his son again.

### 3.5. Conclusion Session

Session 10 is the conclusion session. The aim is to receive and give feedback, and to discuss the therapeutic process. After singing all the songs, a record and a folder with sheets of all the composed songs, the lyrics of the music ritual, and an overview of their own strengths is provided to the songwriter. Then, memorable moments that are the most helpful or make the songwriter the happiest are brainstormed. The music therapist supports the songwriter to reflect on the role of the songs and to identify their own strengths and achievements during the therapeutic process. The discussion involves the transfer of the therapeutic content to day-to-day living at the time after therapy. The songwriter is further engaged in a discussion about the ways to use their experiences and songs to maintain positive experiences and achieve possible new goals in the future. 

## 4. Discussion

In this intervention program, the success and positive emotions caused by creating songs must be ensured by the music therapist. If the songwriter’s intention to work on obtaining positive emotions fails, they will collect more experience of failed efforts [40] and thus might be less motivated to work on their individual symptoms of the depression. In an ongoing study examining the feasibility of the program, it was observed that the music therapist’s contribution to finding positive experiences increased with the severity of the depression. The contribution was facilitated by knowing the songwriter’s biography and carefully observing their activities and environment. In the songwriting process, at least parts of the song must be finished within single sessions. Therefore, adherence to traditional songwriting techniques should be flexible regarding rhymes, metaphors, repeated motives, etc.

We recommend scheduling the ten intervention sessions consistently (for example, one session per week). However, taking real-life practicalities into account, flexibility may be required owing to institutional factors (such as employee sick leave) and individual factors (such as grief, medical appointments, or personal conflicts). Whenever an issue arises that needs to be prioritized, the intervention can be interrupted to accommodate the participant’s needs.

Older adults need sufficient communication skills to write a song. This includes sufficient cognitive skills but also the use of a well-known language within the therapy to prevent a misinterpretation of the songwriter’s statements [20]. Furthermore, the older person needs good cognitive skills for the reflection exercises so they can work with the music therapist to transfer their findings into the lyrics. This might include people with a mild cognitive impairment, but excludes people with significant cognitive impairments, such as dementia. 

In the case of a woman with mild dementia, the music therapist recognized early in the intervention program that her cognitive abilities were insufficient for participating in the songwriting program. To enable songwriting, the music therapist used knowledge from her biography and the activities of the residential care facility to create a song parody for and with her. Applying the song parody technique can simplify the songwriting process. However, using known songs can change the feeling and memories evoked by the song by adding new lyrics. In addition, a song parody may be too abstract for people with dementia. New lyrics to a known song may feel wrong to them or lead to a loss of security. Further songs were not written with the woman, but the regular music therapy continued. In the following weeks, the woman still remembered the song and sang it with the music therapist. Further studies can investigate how writing a ‘Gratitude song’ and a ‘Flourishing song’ can be adjusted for people living with dementia. This might include using songwriting techniques that are more guided by the music therapist or by the structure of a known song.

Within the intervention program, it is important that the proposed songwriting technique matches each participant’s unique interests and their musical and cognitive abilities. For example, a musician might not be interested in a song parody and may prefer to write their three songs using the original songwriting technique. On the other hand, musical skills may be less acute in older age and the songwriter must cope with the loss that can be triggered within the therapeutic songwriting process. In an ongoing feasibility study, an opera singer reported sadness due to the loss of her singing voice. She could not cope with her loss and withdrew from this songwriting project.

Different aspects of the songwriting program interconnect. For example, gratitude might be reported when writing the ‘Pleasure song’, or experiences that evoke pleasure might be reported in the ‘Gratitude song’. Conversely, working on the ‘Gratitude song’ can evoke sadness, particularly if the gratitude is for someone who supported the songwriter during a crisis or is deceased. Empathic support from the music therapist is important to help the songwriter cope with emerging feelings of sadness or anger.

Addressing a spiritual figure was one extension of the original positive psychology intervention ‘Gratitude visit’. In different cases, belief in a spiritual figure was very important to feel grateful. The ‘Gratitude song’ could be extended further to address animals. In the author’s clinical work, a woman wrote the song for her dog who accompanied her for many years when other people around had left. She sang and listened to the song until the day she died.

The therapeutic songwriting program aims to be an extension of the traditional work on depression and to focus on the individual resources and potentialities of the songwriter. Therapeutic songwriting and positive psychology can switch the focus back to an awareness of the developed strengths and resources, especially in older age, where fragility and daily challenges are growing [41]. However, a strong focus on positive emotion without considering negative experiences and their implications can have negative effects. Avoiding negative aspects hinders the development of skills to cope with difficult and sad experiences [42]. To improve wellbeing, a holistic model is needed that considers both positive and negative states. Negative emotions can be highly motivating and can lead to creativity and resilience, as the songwriter is confronted with this and learns to cope with negative experiences [40]. In addition, the learned behavioral mechanism of coping with negative situations can develop character traits [43]. In the intervention program presented here, the recognition of negative experiences is partly integrated in the ‘Gratitude song’ and ‘Flourishing song’. Other people can be of help in coping with challenging situations. In previous applications, the meaning of other people in experiencing pleasure and gratitude was observed regularly. Further, developed strengths and achievements can result from the challenging situations in which the individual finds solutions. A holistic therapeutic approach is possible when the intervention program is complementary to standard care, such as traditional behavior therapy or verbal psychotherapy.

This intervention program was developed for application in Germany. It had previously been successfully applied in an ongoing feasibility study in residential care facilities to investigate its impact on depressive symptoms and wellbeing. Further comprehensive studies are needed to test the effectiveness of the program for people living with late-life depression.

Different components of the program parts, the ‘Pleasure song’, ‘Gratitude song’, and ‘Flourishing song’, could be examined separately to assess its effectiveness on depressive symptoms and wellbeing. Furthermore, an evaluation of the benefits attributed to therapeutic songwriting interventions compared with positive psychology interventions (rather than combined) would help to discern the mechanisms responsible for change and any synergistic effects.

The therapeutic songwriting program was strongly influenced by positive psychology, which is especially effective in western culture [44]. The application in other contexts must be adapted and tested. 

Some elements of the program have been applied in therapies with people with different diagnoses and ages. Interventions involving positive psychology [45] or music therapy [46,47,48] have demonstrated effective reductions in anxiety among adults and older people with various health conditions. As an extension to the current protocol, future research might examine the effects of the program on anxiety and with people who experience other health conditions such as dementia and cancer, or with critically ill patients.

## 5. Conclusions

This intervention program meets the need to identify and develop new treatment possibilities for older people living with late-life depression. Two treatment methods that have already showed significant improvements of depressive symptoms and wellbeing were combined to achieve the best possible effects. However, further research is needed to validate the program and extend the target groups.

## Figures and Tables

**Table 1:** Overview virtues and strengths.

**Wisdom and Knowledge**	**Courage**	**Humanity**
Creativity Curiosity Judgement/Critical thinking Love of learning Perspective	Bravery Perseverance Honesty Zest	Love Kindness Social Intelligence
**Justice**	**Temperance**	**Transcendence**
Teamwork Fairness Leadership	Forgiveness Humility Prudence Self-regulation	Appreciation of Beauty and Excellence Gratitude Hope Humor Spirituality

## Data Availability

Data is not available for this research.
